# Histopathological and immunohistochemical diagnosis of infectious bursal disease in poultry birds

**DOI:** 10.14202/vetworld.2015.1331-1339

**Published:** 2015-11-24

**Authors:** J. Singh, H. S. Banga, R. S. Brar, N. D. Singh, S. Sodhi, G. D. Leishangthem

**Affiliations:** Department of Veterinary Pathology, College of Veterinary Science, Guru Angad Dev Veterinary and Animal Sciences University, Ludhiana, Punjab, India

**Keywords:** histopathology, immunohistochemical, infectious bursal disease

## Abstract

**Aim:**

The aim of the present study was to diagnose infectious bursal disease (IBD) using gross, histopathological, and immunopathological approaches and to compare efficacy of immunohistochemical techniques with conventional diagnostic techniques.

**Materials and Methods:**

A total of 33 samples were collected from the six different poultry farms from Ludhiana and the nearby districts. Upon gross analysis of the necropsied birds, the relevant tissue samples such as bursa, kidney, junction of proventriculus and gizzard, heart, and muscles were then processed for histopathological and immunohistochemical studies.

**Results:**

Varied macroscopic changes were noted in bursa, characterized as swollen, hemorrhages to atrophy in size. Nonetheless, hemorrhages over thigh muscles were rarely seen. Histologically, the bursa showed prominent fibrotic and atrophic changes. Rarefaction of bursal follicles with intermittent infiltration of lympho-mononuclear cells with chronic cystic changes was additional changes, considered to be paramount for IBD. Expression and localization of IBD specific viral antigens were noticed mainly intracellular to the rarefied areas of bursal follicle section(s), in conjunction to inner lining of the cystic cavities of affected follicles. In addition, the junction of proventriculus and gizzard, the heart muscle, respiratory ciliated epithelium, and proventriculus also revealed positive expression to IBD virus (IBDV) antigen. Advanced immunopathological techniques, i.e., immunofluorescence further testified the evidence of antigen as positive green signal within affected follicles. Further consideration to the reliability of various techniques employed, positive correlation (r=0.64623) was emerged out with conventional pathological scoring.

**Conclusion:**

It is concluded that the bursa acts as an organ of choice for demonstrating IBDV antigen for specific diagnosis of disease using immunohistochemistry (IHC), and IHC staining is a precise, specific, rapid, and reliable method to demonstrate the IBDV antigen in the altered tissues due to IBDV infection.

## Introduction

Poultry farming has always been an integral component of livestock production. Besides providing egg and meat, it proves to be a good and reliable source of income in rural areas [1]. There are several viral, bacterial, parasitic, and managemental diseases of poultry that cause direct financial loss to farmers. Among these, infectious bursal disease (IBD) ranks high.

IBD, also known as Gumboro disease [2], based on the area of its first identification (in Gumboro, Delaware United States of America, USA); avian nephrosis [3] and avian infectious bursitis, is classified as an economically important disease of poultry. It is caused by a virus that is member of genus Avibirnavirus of family Birnaviridae with two serotypes of this virus, which have been recognized so far, i.e., serotype 1 and serotype 2 of which serotype 1 is considered pathogenic [4]. On account of the high mutation rate of the IBD virus (IBDV) genome, the virus changes its properties such as antigenic variation with increased virulence [5].

The disease manifests as acute and subclinical form(s) in chicks of age 0-3 weeks as immunosuppression or also in clinical form depending on the age of the bird. The chicks become anorectic, become reluctant to move, and show ruffled feathers with watery diarrhea, trembling and severe prostration. The characteristic gross lesions of the disease include dehydration of the muscles with ecchymotic hemorrhages, enlargement, and orange discoloration of kidneys. The bursa of Fabricius becomes enlarged and shows pale yellow discoloration. Intra-follicular hemorrhages may be found and pin point hemorrhages on the skeletal muscles are usually prominent [6].

The acute phase of the disease lasts for 6-10 days and is characterized by atrophy of bursa along with depletion of B-cells [7] in bursal follicles, the other lymphoid organs such as spleen and cecal tonsils are also affected. Immunosuppression occurs in clinical and subclinical form where both humoral and cellular immune responses are compromised and thus making birds more vulnerable to other secondary infection(s) and reduced response to vaccination [8].

Due to this, IBD is a major threat to the poultry industry both at the national and international level; as evidenced by the concluded submission of Farooq *et al*. [9], who calculated losses of Rs. 4523.99±447.56/- and Rs. 18,276.96±2388.91/- as amount of rupees loss per flock and per year for 1000 broilers, respectively, due to IBD in Mirpur and Kotli districts of Kashmir. The potential for economic losses in the poultry industry also exists when anytime a new antigenic or pathogenic strain of IBDV is introduced into a country or geographic region [10]. Internationally, IBD is also reported endemic in certain areas [11-13]. A total monetary loss of over three billion Nigerian currency was reported by Musa *et al*. [14] over a period of 3-year recurrent outbreaks during years 2009, 2010, and 2011. List of OIE, 2015 [15], has included the disease in the list of notifiable diseases.

Thus, regular vaccination and adoption of proper prophylactic measures are mandatory to diminish the occurance of this disease. In this context, study by Fantay *et al*. [16] showed that the proper time for administration of the vaccine is 18 days post hatch with the management conditions in place at the farm. As this disease has been focused in recent reviews regarding its causative agent [17] and the vaccination [18,19], the present study is, therefore, mainly inclined toward the timely and reliable diagnosis of this threatening disease using gross, histopathology (HP), immunohistochemistry (IHC), and immunofluorescent methods.

## Materials and Methods

### Ethical approval

The present study was conducted after the approval of the research committee and the Institutional Animal Ethics Committee.

### Collection and processing of samples

A total of six poultry farms were visited in and around the areas of Ludhiana and the other districts of Punjab, as shown in Table-1. Prior to the visits to various farms as mentioned, necessary permission was sought with regards to collection of samples from diseased birds and generation of data either from the competent authority of university administration (Guru Angad Dev Veterinary and Animal Sciences University [GADVASU]) as well as from owners of respective farms.

**Table-1:** Various farms visited for sample collection for suspected IBD outbreaks.

S. no.	District of Punjab	Number of samples collected	Total birds	Birds affected (~%)	Age group of affected group (weeks)
Farm I	Khanna, Ludhiana	15	20,000	4	6
Farm II	Chaukiman village	2	16,000	8	4
Farm III	Fatehgarh Sahib	4	100,000	6	8
Farm IV	Gauhar, Ludhiana	7	25,000	10	10
Farm V	Kot Gangurai, Ludhiana	4	10,000	4	3
Farm VI	Moga	1	Backyard farming	2 died out of 10	Variable
Total		33			

IBD=Infectious bursal disease

The mortality recorded in these farms ranged from 3% to 18%. Morbidity was although high. Stress was put on considering only the representative samples from each outbreak. 33 birds suspected for disease were necropsied and gross lesions were noted. The relevant representative tissue samples such as bursa, kidney, junction of proventriculus and gizzard, and heart were subsequently collected in 10% neutral buffered formalin.

The tissues were processed and the 4 µ thick tissue sections were cut out of the paraffin embedded tissue blocks and stained with hematoxylin and eosin staining as per the protocol of Bancroft and Gamble [20] for routine HP.

### IHC

For IHC studies, section(s) were taken on poly-L-Lysine coated slide and then subjected to clearing and then rehydration. The antigen retrieval was carried out in citrate buffer using EZ-Retriever^®^ (Biogenex, USA). These slides were then washed in phosphate buffer saline for 20 min. Serum blocking was done using normal goat serum and subsequently non-specific binding and endogenous peroxidase blocking was followed by overnight incubation with chicken polyclonal to IBDV (Abcam, United Kingdom). It was then followed by 20 min incubation with horseradish peroxidase-conjugated goat polyclonal secondary antibody to chicken IgY-Fc (Abcam, UK). Color was developed with substrate diaminobenzidine (vector) and counterstained with Mayer’s hematoxylin stain. Omission of primary antibodies was used for negative control.

### Immunofluorescence technique

For IHC-fluorescent, tissue sections on poly-L-Lysine coated slides were deparaffinized and rehydrated, and antigen retrieval was done in citrate buffer solution. Blocking of non-specific protein binding and endogenous peroxide was followed by overnight incubation in primary antibody (Chicken polyclonal to IBDV, Abcam, UK). It was followed by incubation with fluorescein isothiocyanate-conjugated goat polyclonal secondary antibody to chicken IgY-Fc (Merck, India) for 20 min in the dark. Counterstaining was done using diamino phenylindole (5 mg/ml, Sigma, USA) for 10 min and after washing mounted in an aqueous glycerol mounting media. Omission of primary antibodies was used for negative control. The slides were viewed under fluorescent microscope (Nikon eclipse).

The antibody was standardized at the dilution of 1:200 for the above two techniques.

### Statistical analysis

The Graphpad Prism software was employed to statistically ascertain the relationship between the histopathological changes in the bursas from affected birds and the immunohistopathological scoring of the histopathologically positive samples. This immunohistopathological scoring was done using the scoring scale of 0-3, as applied by Oladele *et al*. [21] where, three sites in each tissue section were observed under the microscope and scored.

## Results and Discussion

A total of 33 cases during 6 outbreaks were collected, of which 26 were suspected to be having affected with the disease. It is worth to mention here that the stress was put on considering only the representative samples from each outbreak, although the number of the birds affected was more.

### Gross

**Figure-1:**
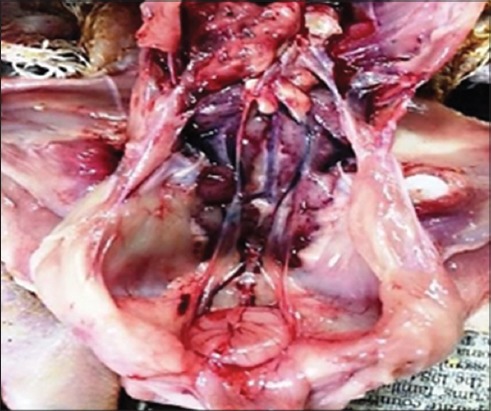
Grossly enlarged and hemorrhagic bursa. The kidneys are also enlarged and hemorrhagic in bird affected with infectious bursal disease.

The bursa of Fabricius in the affected birds aged 4-8 weeks were found showing significant lesions in the birds, which were necropsied in five different farms and showed the atrophic changes. These observations are in accordance with those reported by Juranova *et al*. [22] and Khan *et al*. [23], while in the sixth farm most of the necropsied birds were found to have an enlarged and hemorrhagic bursa (Figure-1). Besides the bursa, the junction of proventriculus and gizzard were also hemorrhagic, as found by Islam and Samad [24]. Renomegaly with congestion and hemorrhages (Figure-1) was noticed in most cases which coincided with the finding of Khan *et al*. [23]; however, the hemorrhages on thigh muscles were scarcely found in three cases. Haghighi *et al*. [5] also reported the similar lesions as hemorrhages in cecal tonsils, hyperemia of the thymus, bursal edema, and hyperemia with enlarged and hyperemic kidneys in gross examination of the experimentally IBDV infected birds.

### HP

**Figure-2:**
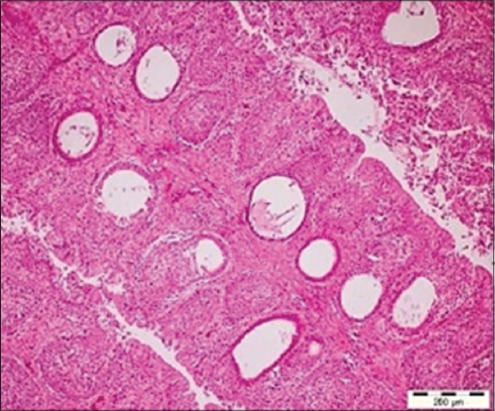
Cystic changes evident in the medullary region in the bursal follicles (H and E, ×10).

The histopathological changes in the bursa of Fabricius mainly showed the fibrotic and atrophic type of changes, which were in the consonance with the gross observations, in which the bursa were found atrophic. Histopathologically, there was rarefaction of the bursal follicles along with the mixed cellular infiltration in the bursal follicles. The cystic changes in the bursal follicles (Figure-2) were also evident in few cases. However, these findings were not in agreement to the findings of Rosenberger [25] and Sharma *et al*. [26], who reported absence of any serious inflammatory bursal lesions. The desquamation and sloughing of bursal epithelium was a frequent finding, which led to erosion of the bursal epithelium. Sellaoui *et al*. [27] also described similar degenerative changes of the coating epithelium.

**Figure-3:**
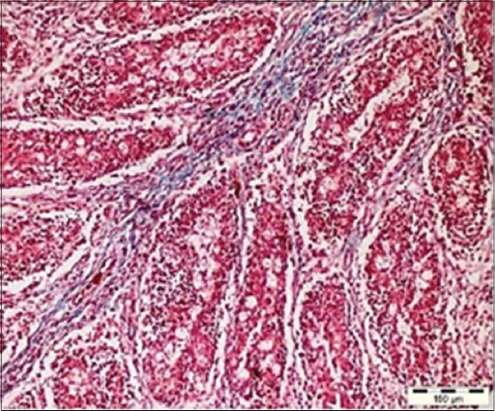
Ratification of the follicular region in the bursa along with the inter-follicular fibrosis (Masson trichrome, ×20).

The grossly atrophied bursa(s) exhibited the fibrosis in between the bursal follicles as evidenced by the fibrous tracts running between them (Figure-3) that was evidenced by pseudolobulation in histopathological examination. Hemorrhages in the bursa along with the development of cystic cavities were quite discernible either in bursal follicles and/or in the bursal epithelium. The presence of cystic cavities along with the cellular accumulation in the bursal follicles was also reported by Guvenc *et al*. [28], whereas Murmu *et al*. [29] reported bursal lesions in vaccinated chickens, which were histopathologically characterized as either normal follicles with or without mild to moderate lymphoid depletion and without follicular atrophy or with the development of cystic follicles. In the present study, some of the bursal follicles showed the degeneration of the core of the follicles along with the presence of pink stained fluid in some of the degenerating follicles. Typical histopathological lesions of the bursa of Fabricius was found similar to as reported by Hoque *et al*. [30], Islam *et al.*, [31] and Rudd *et al*. [32] that included mild to severe lymphoid depletion in bursal follicles, follicular atrophy, cystic formation of follicles, and bursal hemorrhage, but the hemorrhages in bursa were not seen in majority of the cases which was attributed to the collection of samples at the time of post-peak period of occurrence of outbreak, when majority of bursas had already undergone atrophy. Ignjatovic *et al*. [33] reported widespread acute lymphoid necrosis, follicular hemorrhage and stromal edema, indicative of acute IBD, the main findings reported by the scientists were lacking in the present study (follicular hemorrhage and stromal edema),which is an innuendo to the chronic nature of the present outbreaks.

The scoring of the various IBD lesions in bursa of Fabricius was done, as shown in Table-2, based on the criterion and method as adopted by previous researchers such as Moraes *et al*. [34].

**Table-2:** The scoring of the bursal lesions in six outbreaks.

Case no.	Lymphoid depletion	Fibrosis	Cystic spaces	Edema	Congestion/hemorrhage	Proventriculitis
OUTBREAK 1						
JB02A	2	0	0	0	2	2
JB05B	3	2	0	0	0	2
JB08A	2	3	0	0	2	0
JB10A	3	2	0	2	0	0
JB11A	3	3	4	2	2	2
JB13B	3	3	0	0	0	0
JB17C	3	4	0	0	0	0
JB18A	3	2	2	0	0	3
JB19A	3	2	0	0	0	0
JB10B	2	0	2	0	0	2
JB22B	4	3	0	0	1	2
JB23B	3	2	0	0	0	0
JB25b	3	4	0	0	0	0
JB27	2	2	0	0	0	0
	2.785±0.579	2.285±1.20	0.571±1.22	0.285±0.0726	0.5±0.855	0.929±1.141
OUTBREAK 2						
JB34	4	3	0	0	0	0
OUTBREAK 3						
JB39B	3	3	2	0	0	2
JB40	0	0	0	0	0	2
JB41	0	0	0	0	0	2
JB42	0	0	0	0	0	2
	0.75±1.5	0.75±1.5	0.5±1			2±0
OUTBREAK 4						
JB50B	3	3	0	0	2	0
JB50C	2	2	0	0	0	0
JB51B	4	4	0	0	0	0
JB51C	2	2	0	0	0	0
JB52	2	2	0	0	0	0
JB53B	4	3	0	0	0	0
JB53A	3	2	0	0	0	0
	2.86±0.899	2.57±0.786			0.285±0.756	
OUTBREAK 5						
JB62A	4	3	3	3	0	2
JB62B	3	3	2	0	2	2
JB63A	4	4	0	0	0	2
JB65	0	0	0	0	0	2
	2.75±1.89	2.5±1.73	1.25±1.5	0.75±1.5	0.5±1.0	2±0
OUTBREAK 6						
JB71A	2	2	0	0	0	0

As per the analysis of the bursal lesions in these six different outbreaks, the lymphoid depletion was seen the highest in the fourth outbreak (2.86±0.899), followed by fifth (2.75±1.89), and first (2.785±0.579) outbreak, while it was least in the third outbreak (0.75±1.5). In the similar way, the extent to which bursas got fibrosed was also highest in the fourth outbreak (2.57±0.786), but unlike lymphoid depletion, it was followed by fifth (2.5±1.73) and then first outbreak (2.285±1.20). The presence of cysts was highest in the fifth outbreak (1.25±1.5) followed by first (0.571±1.22) and third (0.5±1). On statistical analyses of the extent of lymphoid depletion, fibrosis, cystic spaces, and congestion/hemorrhage in the first and third outbreaks, it is proposed that these four histopathological changes follow a parallel trend in the pathogenesis of the disease. The extent of proventriculitis although followed unrelated trend to these four changes in bursa, it was not only concomitantly found in outbreaks which were marked by extensive bursal histopathological changes, i.e., first (0.929±1.141) and fifth (2±0), but also in the outbreak that exhibited minimal bursal lesions, i.e., outbreak three (2±0). Thus, it is suggested that the IBDV act as a contributor to proventriculitis, rather than being solely responsible for the same. The association of proventriculitis with IBD found in three out of six studied outbreaks goes in accordance with the previous findings by Pantin-Jackwood and Brown [35] and Grau-Roma *et al*. [36], who also reported that naturally occurring proventriculitis can occur in the absence of IBDV and that the IBDV strains tested do not directly produce proventriculitis.

The section(s) from kidney(s) showed marked congestion in the cortex and the medullary area along with the vacuolar tubular degeneration. The kidney showed eosinophilic fluid accumulation, suggesting the proteinaceous nature of fluid along with the lymphomononuclear infiltration in the interstitium and glomeruli. The cerebrum revealed congestion along with the mild perivascular lymphomononuclear cellular cuffing around the blood vessel. The lungs revealed congestion and desquamation of the epithelia of parabronchi. The lungs here showed the infiltrative changes in the parabronchi leading to its thickening.

The liver samples of most of the birds from this outbreak were normal except for some showing some degenerative changes as evidenced by congestion and perivascular cellular infiltration. It was also interesting to find that the liver(s) from one outbreak to be specifically associated with excessive severe fatty change with lymphoid cellular infiltration was seen as aggregate, this further suggests the involvement of mycotoxin-induced injury putatively to be attributed to immunosuppressive effect [37]. The liver was also found hyperemic with the round edges by Haghighi *et al*. [5].

### Immunohistopathology

**Figure-4:**
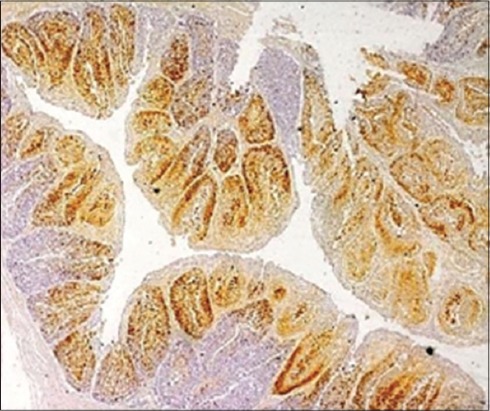
The affected bursal section showing infectious bursal disease viral antigens in the lesion sites identified in bursal follicles (Immunohistochemistry, ×4).

The affected bursal sections revealed viral antigens in the lesion sites identified in tissue sections, especially inside the cells in the rarefied areas of bursal follicles along with the inner lining of the cystic cavities in affected follicles. In various previous experimental studies, IBDV antigens have been detected in macrophages within follicles, the interstitium, and the lymphoid cells of bursa of Fabricius, e.g., by Oladele *et al*. [21], but in the present study, the viral antigens were found mainly associated with bursal lymphoid cells and in bursal epithelium in some cases (Figure-4). The findings can be related to the findings of Tippenhauer *et al*. [38] who reported higher IBDV antigen load in bursa of Fabricius of layer type birds, in addition to the clinical signs and death rate when compared to the broiler type birds, in their differential immunopathogenic study. The coinciding fact in the present study is being the consideration of mainly layer type birds. Nunoya *et al*. [39] and Tanimura *et al*. [40] detected antigen in cytoplasm of lymphocytes, epithelial cells, and inflammatory cells (mostly macrophages) in the bursa of Fabricius of IBDV infected chickens, but Jonsson and Engstrom [41], however, observed antigen only in bursal lymphocytes, which goes in exact accordance with the findings of the present study. In addition to this, the findings of the present study also coincided with the findings of Haghighi *et al*. [5] in context to the viral antigen distribution within the cells of different tissues, where the viral antigens were found dispersed as fine to coarse granules within the infected or degenerated cells.

**Figure-5:**
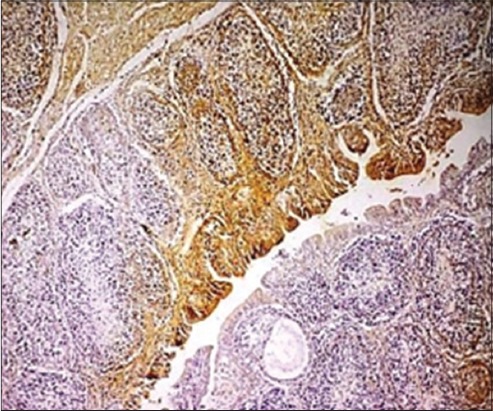
Bursal interfollicular connective tissue as well as the bursal epithelium showing the presence of infectious bursal disease viral antigen (Immunohistochemistry, ×20).

**Figure-6:**
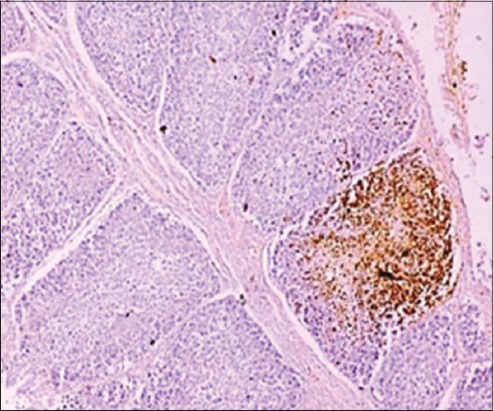
A bursal section demonstrating the focally extensive localization of infectious bursal disease viral antigens (Immunohistochemistry, ×40).

**Figure-7:**
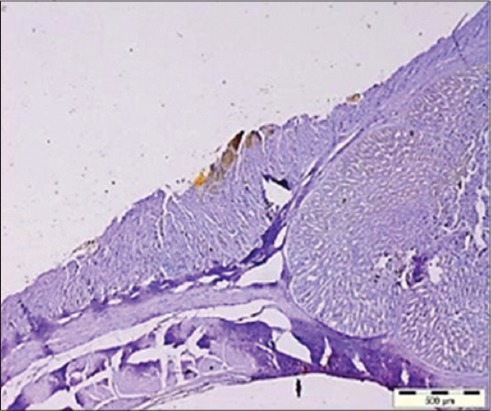
The junction of proventriculus and gizzard expressing the infectious bursal disease virus antigens (Immunohistochemistry, ×4).

**Figure-8:**
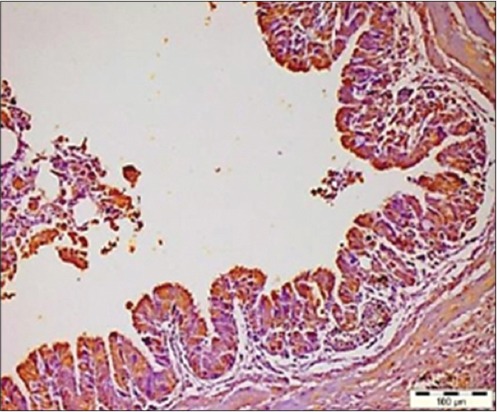
Respiratory ciliated epithelium showing the infectious bursal disease virus antigens (Immunohistochemistry, ×20).

**Figure-9:**
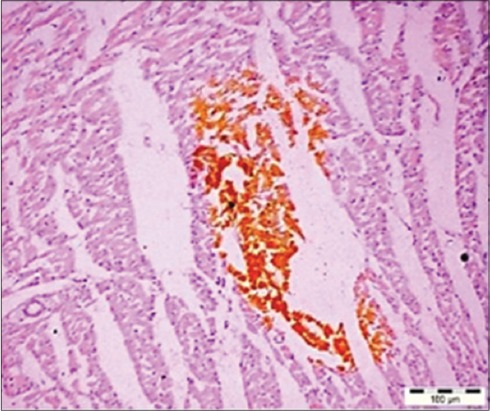
The infectious bursal disease virus antigen in the heart muscles (Immunohistochemistry, ×20).

The presence of viral antigen was also appreciated in the bursal interfollicular connective tissue (Figure-5) as well as the bursal epithelium which was also reported by Oladele *et al*. [21]. The distribution of viral antigen varied from being localized (Figure-6) to focal to diffuse. In addition, the junction of proventriculus and gizzard (Figure-7) was also found to be expressing the IBDV antigen. The IBDV antigens were also localized in the respiratory ciliated epithelium (Figure-8) at the affected sites in the proventricular sections and in the heart muscle (Figure-9), whereas Pantin-Jackwood and Brown [35] did not report any IBDV antigen in the proventriculus. Hemalatha *et al*. [42] also reported the similar IHC results in 100 1-day-old experimentally infected white leghorn male chicks, where the reaction of varying intensities was seen in isolated lymphoid cells in cortex and surface epithelium of bursa of Fabricius. The similar reaction was also seen in the cortical lymphocytes of thymus and in few glandular epithelial cells of Harderian gland depending on the days post-infection.

**Figure-10:**
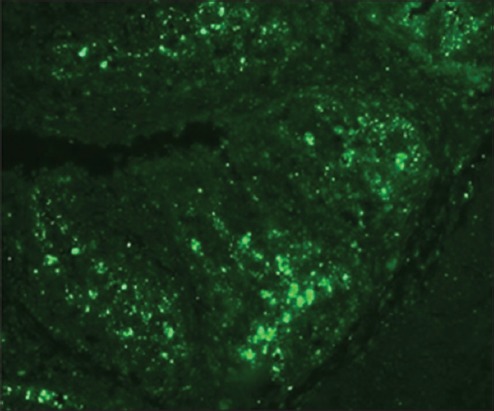
Infectious bursal disease virus antigen in the cystic lesions in bursal follicles (IFT, ×20).

Besides this, the immunofluorescent technique was also employed that further confirmed/supplemented the presence of IBDV in the cystic lesions in bursal follicles (Figure-10), which was in consonance with the findings of Mahgoub [17] and Van den Berg *et al*. [43]. In addition, Hemalatha *et al*. [42] detected the viral antigens by specific fluorescence in lymphoid cells, plical epithelium, macrophages in bursa of Fabricius, and Kupffer cells in liver and proventricular glandular epithelial cells.

### Statistical analysis

To statistically ascertain the relationship between the histopathological changes in the bursas from affected birds and the immunohistopathological scores, the immunohistopathological scoring was done using the scoring scale of 0-3, as applied by Oladele *et al*. [21], as shown in Table-3. For a statistical comparison to be drawn, the mean and the standard deviation of the respective histopathological changes were also calculated, as shown in Table-4. This mean represented the overall damage to the histo-architecture, which was then compared with the IHC scoring of the 27 histopathologically positive samples (out of 33 total samples) using the Graphpad Prism software.

**Table-3:** Immunohistopathological scoring criterion.

IHC positive cell (per×40 objective lens of the light microscope)	Score
<5 stained cells	0
5-50 stained cells	1
50-150 cells	2
Over 150 stained cells	3

IHC=Immunohistochemistry

**Table-4:** Overall histopathological scoring and the IHC scoring, as per Oladele *et al.*[21].

Slide no.	Lymphoid depletion	Fibrosis	Cysts	Edema	Congestion	Mean	Standard deviation	IHC score
JB02A	2	0	0	0	2	0.8	1.095445	0
JB05B	3	2	0	0	0	1	1.414214	0
JB08A	2	3	0	0	2	1.4	1.341641	2
JB10A	3	2	0	2	0	1.4	1.341641	2
JB11A	3	3	4	2	2	2.8	0.83666	3
JB13B	3	3	0	0	0	1.2	1.643168	3
JB17C	3	4	0	0	0	1.4	1.949359	2
JB18A	3	2	2	0	0	1.4	1.341641	1
JB19A	3	2	0	0	0	1	1.414214	0
JB10B	2	0	2	0	0	0.8	1.095445	0
JB22B	4	3	0	0	1	1.6	1.81659	3
JB23B	3	2	0	0	0	1	1.414214	2
JB25b	3	4	0	0	0	1.4	1.949359	2
JB27	2	2	0	0	0	0.8	1.095445	2
JB34	4	3	0	0	0	1.4	1.949359	3
JB39B	3	3	2	0	0	1.6	1.516575	3
JB50B	3	3	0	0	2	1.6	1.516575	2
JB50C	2	2	0	0	0	0.8	1.095445	0
JB51B	4	4	0	0	0	1.6	2.19089	3
JB51C	2	2	0	0	0	0.8	1.095445	1
JB52	2	2	0	0	0	0.8	1.095445	2
JB53B	4	3	0	0	0	1.4	1.949359	2
JB53A	3	2	0	0	0	1	1.414214	2
JB62A	4	3	3	3	0	2.6	1.516575	3
JB62B	3	3	2	0	2	2	1.224745	3
JB63A	4	4	0	0	0	1.6	2.19089	2
JB71A	2	2	0	0	0	0.8	1.095445	2

IHC=Immunohistochemistry

**Figure-11:**
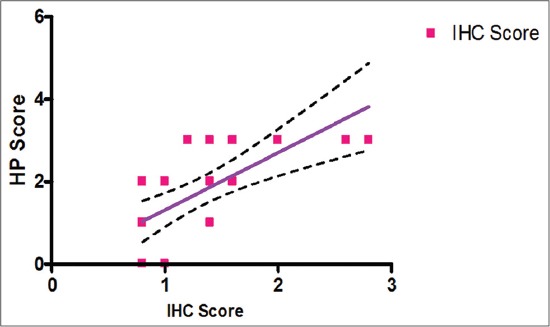
Correlation between histopathology (HP) score and immunohistochemistry (IHC) score.

The correlation coefficient between the mean histopathological scoring and the IHC scoring was also found to be positive, 0.64623 for the respective cases, which proves the reliability on IHC for the positive cases. Further, it can also be represented graphically, as depicted in Figure-11, where both of the curves follow similar trend: At 95% confidence interval, the slope: 0.1219-0.4220, with R²=0.3580 and at p<0.001, as shown in Table-5.

**Table-5:** Correlation between HP score against immunohistopathological score in different samples of IBD.

Pearson’s correlation coefficient (r)	Slope	(95% CI)	p value	R2
0.64623	1.392±0.3288	0.0003	0.4176
	(0.7148-2.069)		

CI=Confidence interval, IBD=Infectious bursal disease, HP=Histopathology

## Conclusion

It is concluded that the bursa is the main organ involved in the pathogenesis of IBD and thus also acts as an organ of choice for demonstrating IBDV antigen for specific diagnosis of disease using IHC. Although IHC method of diagnosis has previously been compared with other methods as well [44], yet here it yielded obvious results. It is concluded that IHC staining is a precise, specific, rapid, and reliable method to demonstrate the IBDV antigen in the altered tissues due to IBDV infection, which is in concurrence with gross or microscopic lesions and supplemented by immunofluorescent technique.

## Authors’ Contributions

JS and HSB initiated research concept and design, collection and analysis of data was compiled by JS, NDS, SS, and GDL. The interpretation of HP was done by HSB and JS. Slide’s photography was done by JS and GDL. HSB and RSB critically reviewed the article, while the final approval of the article was done by JS, HSB, SS, NDS, and RSB.
